# FSD-C10: A more promising novel ROCK inhibitor than Fasudil for treatment of CNS autoimmunity

**DOI:** 10.1042/BSR20150032

**Published:** 2015-09-10

**Authors:** Yan-Le Xin, Jie-Zhong Yu, Xin-Wang Yang, Chun-Yun Liu, Yan-Hua Li, Ling Feng, Zhi Chai, Wan-Fang Yang, Qing Wang, Wei-Jia Jiang, Guang-Xian Zhang, Bao-Guo Xiao, Cun-Gen Ma

**Affiliations:** * Institute of Brain Science, Department of Neurology, Medical School, Shanxi Datong University, Datong 037009, China; † “2011” Collaborative Innovation Center/Research Center of Neurobiology, Shanxi University of Traditional Chinese Medicine, Taiyuan 030619, China; ‡ Department of Neurology, Thomas Jefferson University, Philadelphia, PA 19107, U.S.A.; § Institute of Neurology, Huashan Hospital, Institutes of Brain Science and State Key Laboratory of Medical Neurobiology, Fudan University, Shanghai 200040, China

**Keywords:** Rho kinase, Rho kinase inhibitor, Fasudil, FSD-C10

## Abstract

Rho-Rho kinase (Rho-ROCK) triggers an intracellular signalling cascade that regulates cell survival, death, adhesion, migration, neurite outgrowth and retraction and influences the generation and development of several neurological disorders. Although Fasudil, a ROCK inhibitor, effectively suppressed encephalomyelitis (EAE), certain side effects may limit its clinical use. A novel and efficient ROCK inhibitor, FSD-C10, has been explored. In the present study, we present chemical synthesis and structure of FSD-C10, as well as the relationship between compound concentration and ROCK inhibition. We compared the inhibitory efficiency of ROCKI and ROCK II, the cell cytotoxicity, neurite outgrowth and dendritic formation, neurotrophic factors and vasodilation between Fasudil and FSD-C10. The results demonstrated that FSD-C10, like Fasudil, induced neurite outgrowth of neurons and dendritic formation of BV-2 microglia and enhanced the production of neurotrophic factor brain-derived neurotrophic factor (BDNF), glial cell line-derived neurotrophic factor (GDNF) and neurotrophin-3 (NT-3). However, the cell cytotoxicity and vasodilation of FSD-C10 were relatively small compared with Fasudil. Although Fasudil inhibited both ROCK I and ROCK II, FSD-C10 more selectively suppressed ROCK II, but not ROCK I, which may be related to vasodilation insensitivity and animal mortality. Thus, FSD-C10 may be a safer and more promising novel ROCK inhibitor than Fasudil for the treatment of several neurological disorders.

## Abstract (precis)

Compared with Fasudil, novel Rho kinase inhibitor FSD-C1 exhibited similar therapeutic potential and mechanisms in EAE, but had low cytotoxicity and vasodilation, providing a more promising novel ROCK inhibitor for the treatment of several neurological disorders.

## INTRODUCTION

Rho kinase (ROCK), a serine/threonine kinase, is activated by binding to the active GTP-bound form of the small GTPase Rho. ROCK is expressed both centrally and peripherally where it is implicated in fundamental cellular processes including migration, proliferation and survival [1]. The research in RhoA/ROCK pathway has attracted much attention for more than a decade since the discovery of ROCK in 1996. A series of studies have demonstrated that the blockade of Rho/ROCK is considered to be beneficial for inflammatory demyelination and degeneration in the central nervous system (CNS) and has proved to be efficacious in animal models of stroke [2], multiple sclerosis (MS) [3–6], amyotrophic lateral sclerosis (ALS) [7], Alzheimer's disease (AD) [8] and Parkinson's disease (PD) [9,10]. Therefore, Rho/ROCK pathway is a promising therapeutic target in neurodegenerative and neurotraumatic diseases and ROCK inhibitor should be a promising drug for preventing neurodegeneration and stimulating neuroregeneration in several neurological diseases [11].

Fasudil hydrochloride [hexahydro-1-(5-isoquinolinyl-sulfonyl)-1H-1,4-diazepine monohydrochloride (HA 1077)] is a isoquinoline sulfonamide derivative which is the most commonly used for pharmacological ROCK inhibitor, both for *in vitro* and *in vivo* studies. At present, Fasudil is only used in clinic as a ROCK inhibitor for preventing and improving the cerebral vasospasm after subarachnoid haemorrhage and symptoms of cerebral ischaemia. Previous studies showed that ROCK inhibitor also promotes the survival of neural stem cells, axonal regeneration and differentiation of bone marrow mesenchymal cell into neurons [12,13]. Yamashita and colleagues [14] observed that Fasudil can effect on neurons directly by reducing the activity of ROCK and protect neuronal ischaemic damage in persistent model of cerebral ischaemia.

When Fasudil displays certain beneficial effect, there are many limitations in clinical use, including short-course treatment, low oral bioavailability, cell toxicity and blood pressure fluctuation. Therefore, a considerable interest and efforts have been devoted to the development of novel ROCK inhibitors that should be taken orally for long-term use, with low cytotoxicity and blood pressure fluctuation. We have designed a novel ROCK inhibitor FSD-C10 that exhibits therapeutic potential in experimental autoimmune encephalomyelitis (EAE), an animal model of MS. In the present study, we explored and compared the cell cytotoxicity, neurite outgrowth and dendritic formation, neurotrophic factors, vasodilation and safety between Fasudil and FSD-C10.

## MATERIALS AND METHODS

### ROCK inhibition by mobility shift assay

The inhibition efficiency of Fasudil and FSD-C10 on ROCK activity was measured by mobility shift assay with ATP concentration (Sigma) at 3.6 μM against ROCK I (Carna) and at 5.3 μM against ROCK II (Carna) according to the manufacturer's protocol. Staurosporine (Sigma) was used as positive control and saline was used as negative control.

Fasudil and FSD-C10 were diluted to the final desired highest compound concentration (10 μM) by 100% DMSO and serially diluted on 96-well plate by transferring 30–60 μl of 100% DMSO in the next well for a total of 10 concentrations in duplicate. DMSO (100 μl) was added to two empty wells for no compound control and no enzyme control in the same 96-well plate. Mobility shift assay was performed according to the manufacturer's protocol. Briefly, compound Fasudil and FSD-C10 (10 μl) were mixed with 90 μl of kinase buffer (50 mM HEPES, pH 7.5, 0.0015% Brij-35, 10 mM MgCl_2_, 2 mM DTT) in 96-well plate. The mixtures (5 μl) were incubated with 2.5× enzyme solution (10 μl) in 384-well plate at room temperature for 10 min and control was performed by adding 5 μl of kinase buffer. Substrate solution (10 μl) was added at 28°C and the enzyme reaction was stopped by adding 25 μl of stop solution (100 mM HEPES, pH 7.5, 0.015% Brij-35, 50 mM EDTA) to all wells.

$$\mathrm{Percent}\;\mathrm{inhibition}\;=\;\left(\frac{\max-\mathrm{conversion}}{\max-\min}\right)\;\times \;100$$

where max stands for DMSO control; min stands for low control.

### Primary neuron culture

Embryonic 18-day-mouse cortex (Shanghai SLAC Laboratory Animal Co. Ltd) were dissected under a microscope and then triturated into the suspension in Neurobasal-A-medium (Gibco) supplemented with 2% B27 (Gibco), 100 units/ml penicillin and 100 μg/ml streptomycin (Gibco). The cells were then plated on cover slips coated with poly-D-lysine (0.1 mg/ml; Sigma) at a density of 1×10^6^ cells per cm^2^ at 37°C in a humidified cell incubator under a 95%/5% (v/v) mixture of air and CO_2,_ with the cell media being replaced every 48 h. After 7 days, neurons were incubated with different conditioned medium.

### BV-2 microglial culture

The BV-2 immortalized microglial cell line [15] was purchased from ShenKe Biological Technology Co. and cultured in Dulbecco's modified Eagle's medium (DMEM; Gibco), supplemented with 10% FBS (Gibco), 100 units/ml penicillin and 100 μg/ml streptomycin (Gibco) at 37°C in a humidified cell incubator under a 95%/5% (v/v) mixture of air and CO_2._ Cells were plated on a 96-well plate at a density of 1.0×10^6^ /ml (200 μl/well), on a 24-well plate at 1.0×10^6^/ml (500 μl/well) and at 0.2×10^6^ /ml (500 μl/well) for different experiments.

### Evaluation of cell state

We used two widely accepted assays, lactate dehydrogenase (LDH) release and MTT reduction assay, for the measurement of cell viability and death. These assays are considered reliable and reproducible with high predictive validity and widely used in various pharmacological studies [16].

#### MTT assay

Cell viability of BV-2 microglia and primary neurons was detected by MTT assay. In brief, 100 μl of MTT solution (0.5 mg ml^−1^, Duchefa) was added to cultured cells and incubated for an additional 4 h at 37°C, until the medium turned purple. Absorbance at 570 nm was measured by a microplate reader after addition of 100 μl DMSO. Each experiment was performed in triplicate and repeated three times with separate cell preparations. Results were expressed as a percentage of the control value at 570 nm.

#### LDH assay

The death of BV-2 microglia and primary neurons was measured by LDH release in the culture medium. Levels of LDH release in supernatants of cultured cells were measured using the cytotoxicity detection kit (Promega) according to the manufacturer's protocol. Maximum LDH release (*A*_570_) was determined following the treatment of cells with lysis buffer and considered as control value (100% LDH release). Data were expressed as a percentage of the control value.

### Animals

Female C57BL/6 mice (8–10 weeks old and 18–20 g of body weight) were purchased from Vital River Laboratory Animal Technology Co. Ltd. All experiments were conducted in accordance with the guidelines of the International Council for Laboratory Animal Science. The study was approved by the Ethics Committee of Shanxi Datong University. All mice were housed under pathogen-free conditions and maintained in a reversed 12: 12-h light/dark cycle in a temperature controlled room (25±2°C) for 1 week prior to experimental manipulation.

### Induction and clinical evaluation of EAE

Mouse myelin oligodendrocyte glycoprotein peptide 35–55 (MOG_35–55_, MEVGW YRSPFSRVVHLYRNGK) was produced in an automatic synthesizer (CL. Bio-Scientific Company). The purity of the peptide was >95% as determined by HPLC. Chronic EAE was induced by subcutaneous immunization on the upper dorsal flanks with 300 μg of MOG_35–55_ in Freund's complete adjuvant (Sigma) supplemented with 3 mg/ml of M. Tuberculosis H37Ra (BD Difco; 400 mg/mice). Mice were injected intraperitoneally (i.p.) with 500 ng of pertussis toxin (Enzo Life Sciences) on days 0 and 2 post-immunization (p.i.). Animals were weighed and evaluated for clinical score every other day in a blinded fashion by at least two investigators. Clinical score of EAE was graded according to the following criteria: 0: healthy; 1: limp tail; 2: ataxia and/or paresis of hind limbs; 3: paralysis of hind limbs and/or paresis of forelimbs; 4: tetraparalysis; and 5: moribund or death. When the clinical score of EAE reaches 3, we give suitable care including softening of food with water in dish and additional nutrients such as egg, making mice easy to obtain food and nutrition.

### Administration of Fasudil and FSD-C10

Mice were divided into three groups: Fasudil-treated group (*n*=8), FSD-C10-treated group (*n*=8) and double-distilled water (ddH_2_O) EAE control group (*n*=9). Fasudil or FSD-C10 (from Tianjin Chase Sun Pharmaceutical Co.) was dissolved in sterile ddH_2_O. Mice were i.p. injected with 800 μg/200 μl/daily from day 3 to day 27 p.i. Mice that received the same volume of ddH_2_O i.p. served as control.

### Assessment of neurite outgrowth and dendritic length

Fluorescent photomicrographs of neurons with microtubule-associated protein-2 (MAP-2) staining or photomicrographs of BV-2 cells under inverted microscope were captured with a 20× objective and a digital camera of Olympus microscope. Images were acquired from randomly selected fields for each group. The length of the longest neurite outgrown or dendritic formation in the cell body was measured by an investigator blinded to the experimental groups using a public-domain image-processing program (Image J, http://rsb.info.nih.gov/ij). Average lengths of neuronal neurite or dendritic formation were used for quantification.

### Western blot analysis

Protein from brains was homogenized on ice with an extraction kit and protein concentration was determined by a Bradford protein assay. Protein extracts (30 μg) were separated by SDS/PAGE and transferred on to a PVDF membrane (Immobilon-P; Millipore Corp.). The membranes were then incubated with anti-GDNF (glial cell line-derived neurotrophic factor; 1:1000, Epitomics), anti-BDNF (brain-derived neurotrophic factor; 1:1000, Promega), anti-NT-3(neurotrophin-3; 1:1000, Epitomics), anti-Nogo(1:1000, Epitomics), anti-iNOS (inducible nitric oxide synthase) (1:1000, Cell Signaling Technology) Enzo Life Sciences, anti-arginase-1 (Arg-1) (1:1000, BD Bioscience) and anti-β-actin (1:10000, Cell Signaling Technology) for overnight at 4°C. Bands were visualized by horseradish peroxidase (HRP-) conjugated secondary antibodies and ECL kit under ECL system (GE Healthcare Life Sciences).

### The test of vasodilation

Both Fasudil and FSD-C10 were dissolved in sterile ddH_2_O and the concentration was 4 mg/ml. Naive female C57BL/6 mice were i.p. injected with 200 μl of Fasudil or FSD-C10 and the pictures of limbs were taken at 30 and 60 min after drug injection.

### Statistical analysis

All the experiments were repeated two or three times and GraphPad Prism software was used for statistical analysis. Student's *t*test was performed to analyse the difference between any two groups. A statistically significant difference was assumed at *P*<0.05.

## RESULTS

### Chemical synthesis and structure of FSD-C10

**Figure 1:**
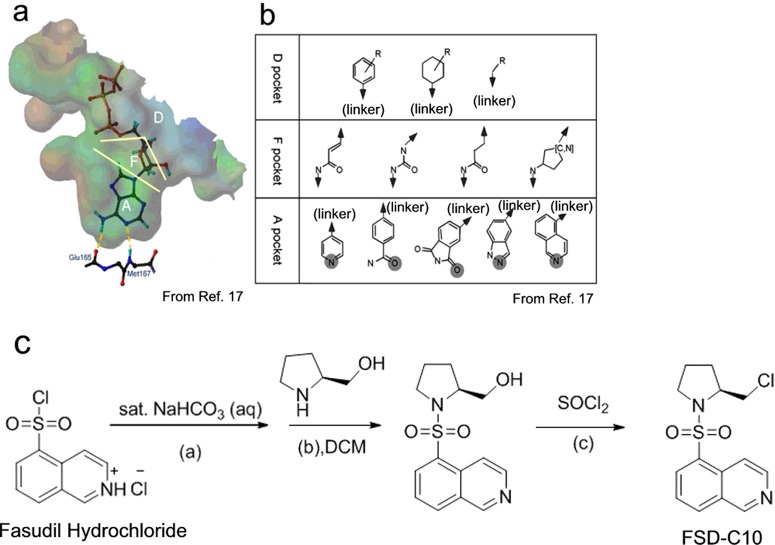
Synthesis of FSD-C10 (**a**) Ligand-binding pocket of ROCK homology model and (**b**) chemical substructures designated for each division of the ligand-binding pocket that are reproduced from [17]: Takami, A., Iwakubo, M., Okada, Y., Kawata, T., Odai, H., Takahashi, N., Shindo, K., Kimura, K., Tagami, Y., Miyake, M. et al. (2004) Design and synthesis of Rho kinase inhibitors. Bioorg. Med. Chem. **12**, 2115–2137. The pocket is divided into three parts: region A, F and D. The pocket D region is cleft-like in shape and a wide range of chemical fragments would fit this cleft [17]. Therefore, we designed and synthesized an isoquinoline FSD-C10 targeting region D of the Fasudil. (**c**) Synthetic process of FSD-C10. (**a**) CH_2_Cl_2_, saturated sodium bicarbonate solution, 0°C. (**b**) CH_2_Cl_2_, Et_3_N, 2 h, 0°C, 95% yield. (**c**) CH_2_Cl_2_, reflux, 7 h, 75% yield.

In 2004, Takami et al. [17] reported a ROCK ligand-binding pocket model that is divided into three parts: region A, region F and region D (Figure 1a). It was speculated that the nitrogen atom of region D of Fasudil on the homopiperazine moiety might form a similar hydrogen bond with ATP, which could improve the bioactivity of the compound. Therefore, we designed and synthesized an isoquinoline ROCK inhibitor FSD-C10 targeting region D of Fasudil as the lead compound and on the basis of ligand-binding pocket theory (Figure 1b).

### The inhibition of ROCK by Fasudil and FSD-C10

**Figure 2:**
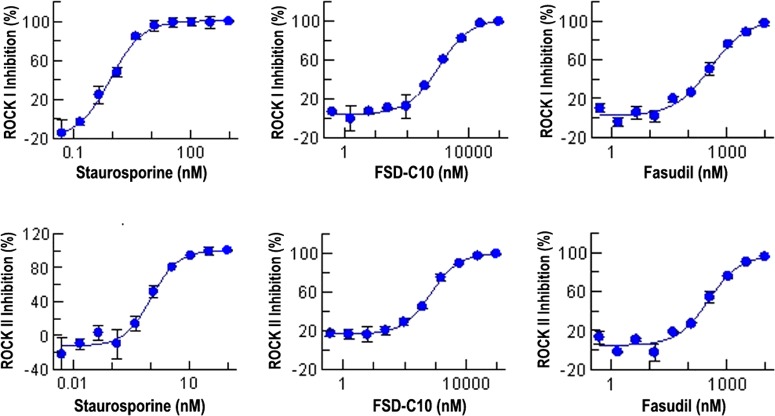
The relationship between compound concentration and ROCK inhibition Fasudil and FSD-C10 were diluted to the final desired highest compound concentration (10 μM) by 100% DMSO and serially diluted on 96-well plate by transferring 30–60 μl of 100% DMSO in the next well for a total of 10 concentrations in duplicate. DMSO (100 μl) was used for no compound control and no enzyme control. Percentage of ROCK inhibition was calculated by 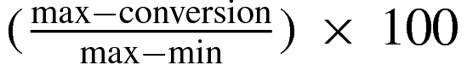. ‘Max’ stands for DMSO control; ‘min’ stands for low control.

Figure 2 shows the relationship between compound concentration and percentage of ROCK inhibition. As shown in Table 1, the inhibition of ROCK activity (IC_50_) was measured with ATP concentration at 3.6 μM against ROCK I and at 5.3 μM against ROCK II and observed in Fasudil (ROCK I=385 nM; ROCK II=344 nM) and FSD-C10 (ROCK I=1141 nM; ROCK II=711 nM) as compared with negative controls (ROCK I > 10000; ROCK II > 10000). However, the inhibitory efficiency of FSD-C10 on ROCK activity was weaker than that of Fasudil, especially in ROCK I activity (Table 1).

**Table 1:** The inhibition efficiency of Fasudil and FSD-C10 on ROCK activity

Compound	Unit (nM)	ROCK I ATP con. (3.6 μM)	ROCK II ATP con. (5.3 μM)
Fasudil	IC_50_	385	344
FSD-C10	IC_50_	1141	711
Positive control	IC_50_	1.7	0.93
Negative control	IC_50_	>10000	>10000

### The therapeutic effect of Fasudil and FSD-C10 in EAE

**Figure 3:**
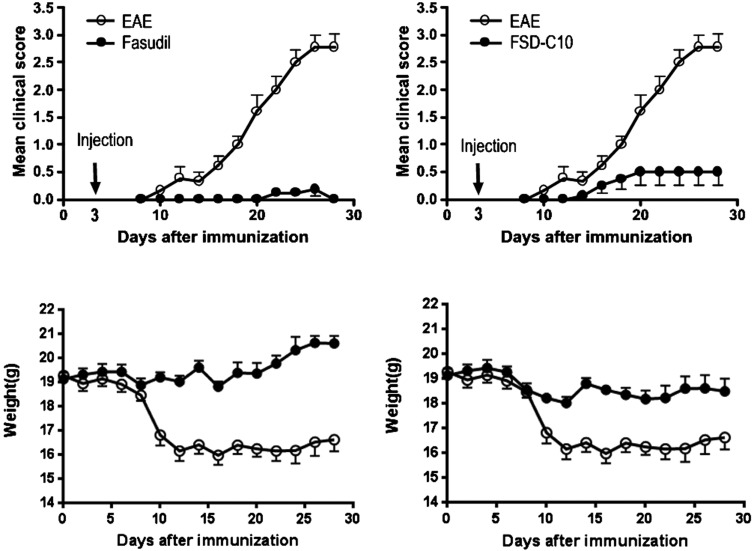
Fasudil and FSD-C10 ameliorated the severity of EAE Chronic EAE was induced in C57BL/6 mice with MOG_35–55_ peptide. Mice received Fasudil or FSD-C10 by i.p. injection from day 3 to 27 p.i. Mean clinical score and mean body weight were recorded. The comparison in each time point was separately analysed by Mann–Whitney U test after non-parametric Kruskal Wallis test.

In the present study, mice were immunized with MOG_35-55_ peptide to develop an EAE model that closely imitates many characteristics of MS. Starting on day 3 p.i., mice received 800 μg/200 μl daily of Fasudil or FSD-C10 until on day 27 p.i. by i.p. injection. As shown in Figure 3, maximum clinical score in EAE control group (*n*=9) was 2.78, mean clinical score was 1.29±1.08 compared with mice injected with Fasudil (*n*=8, maximum clinical score=0.19; mean clinical score=0.04±0.07, *P*<0.001) and FSD-C10 (*n*=8, maximum clinical score=0.50; mean clinical score=0.28±0.23, *P*<0.001). There is a relationship between loss of body weight and severity of clinical score during the development of EAE. The treatment of Fasudil or FSD-C10 had less body weight loss as compared with that of EAE mice (Figure 3, both *P*<0.001).

### The effect of Fasudil and FSD-C10 on cell viability and death

**Figure 4:**
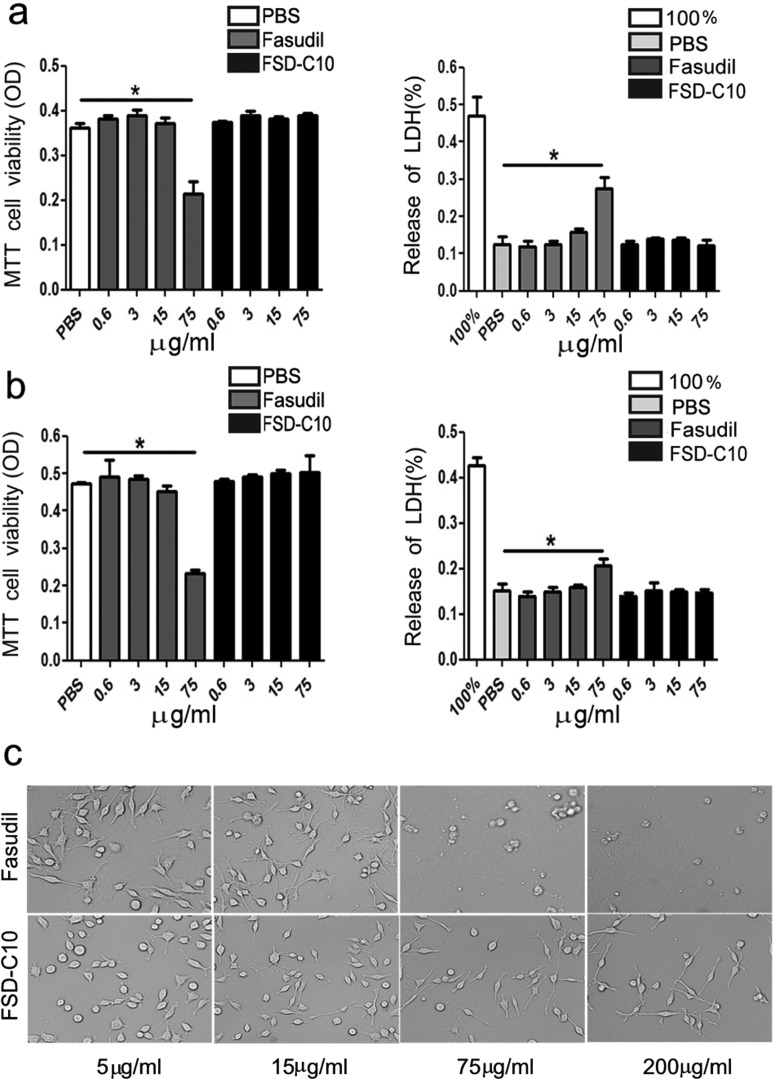
The viability and death of primary neurons (**a**) and BV-2 microglia (**b**) *in vitro* Cell viability was measured using the MTT assay and cell death was detected by LDH release assay. Primary neurons and BV-2 microglia were treated with different concentrations of Fasudil or FSD-C10 (0.6, 3, 15 and 75 μg/ml) for 24 h. The quantitative data are mean±S.E.M. based on three independent experiments with similar results; *P<0.05.

Cell viability and death after culture with Fasudil or FSD-C10 were determined by the MTT and LDH assays (Figure 4). Low/medium-dose Fasudil or FSD-C10 (ranging 0.6–15 μg/ml) did not influence the viability and death of cultured primary neurons, whereas high concentration of Fasudil (75 μg/ml) caused a significant decrease in neuron viability (*P*<0.05) and neuron death (*P*<0.05). In contrast, the same concentration of FSD-C10 did not lead to significant decrease in neuron viability and death compared with control (Figure 4a).

Similarly, low/medium-dose Fasudil or FSD-C10 (ranging 0.6–15 μg/ml) did not influence the viability and death of BV-2 microglia, whereas high concentration of Fasudil (75 μg/ml) caused a significant decrease in BV-2 cell viability (*P*<0.05) and death (*P*<0.05). In contrast, FSD-C10 (75 μg/ml) did not lead to significant decrease in BV-2 cell viability and death compared with control (Figure 4b). As expected, BV-2 microglia treated with higher concentration of Fasudil (75 μg/ml and 200 μg/ml) exhibited obvious cell death after 48 h culture, whereas FSD-C10-exposed cells at similar concentrations showed a good cell morphology (Figure 4c). However, higher doses of FSD-C10 (≥200 μg/ml) also caused cell death with the extension of time (result not shown). The results show that FSD-C10 cytotoxicity is lower than Fasudil in *in vitro* experiments.

### The effect of Fasudil and FSD-C10 on neurite outgrowth of neurons and dendritic formation of BV-2 microglia

**Figure 5:**
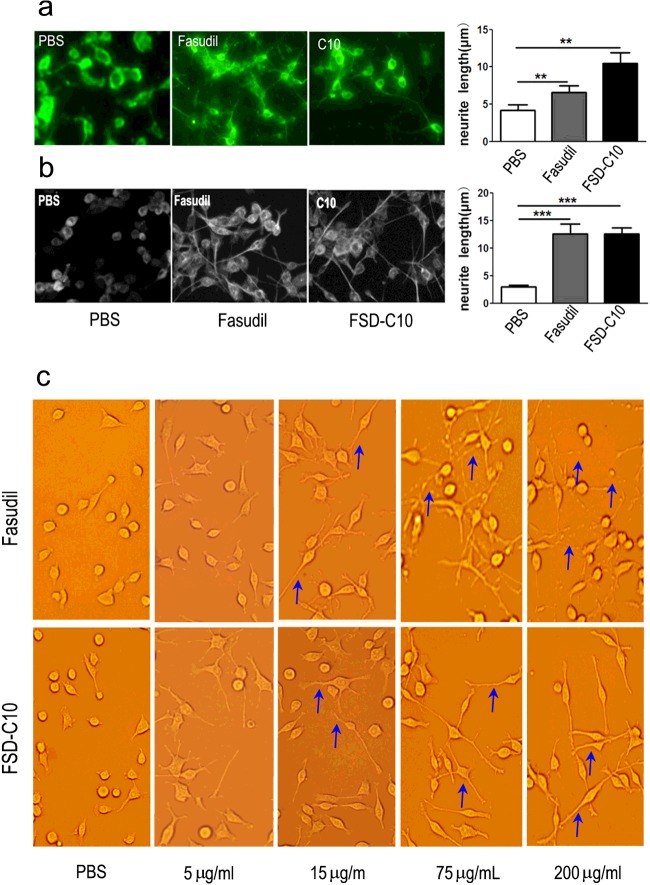
Fasudil and FSD-C10 promoted neurite outgrowth in primary neurons and dendritic formation in BV-2 microglia The neurite length of primary neurons (**a**) and the dendritic length of BV-2 microglia (**b**) was examined with an inverted Olympus microscope. (**c**) High concentrations of Fasudil (75 and 200 μg/ml), but not FSD-C10, caused significant breakage of neurite outgrowth. The length of the longest neurite outgrown or dendritic formation in the cell body was measured by a public-domain image-processing program The quantitative data are mean±S.E.M. based on three independent experiments with similar results; **P<0.01, ***P<0.001.

ROCK has a key role in blocking axon growth and pharmacological ROCK inhibition using small molecules inhibitors has shown to increase axonal regeneration [18,19]. We next explored the effect of Fasudil and FSD-C10 on neurite outgrowth of primary neurons and dendritic formation of BV-2 microglia. As shown in Figure 5(a), the neurite length of primary neurons was significantly prolonged after the treatment of Fasudil (mean=6.6 μm) and FSD-C10 (mean=10.5 μm) as compared with PBS control (4.1 μm, both *P*<0.01).

The dendritic length of BV-2 microglia was also significantly prolonged after the treatment of Fasudil (mean=12.5 μm) and FSD-C10 (mean=12.5 μm) as compared with PBS control (2.94 μm, both *P*<0.001; Figure 5b). These results indicate that FSD-C10, like Fasudil, exhibited the basic characteristics of ROCK inhibitor, contributing to the neurite outgrowth of neurons and dendritic formation of BV-2 microglia. However, high concentrations of Fasudil (75 and 200 μg/ml) caused significant breakage of dendritic branches (blue arrow), whereas FSD-C10, even at the high concentrations (75 and 200 μg/ml), did not cause significant breakage of dendritic branches on BV2 microglia (Figure 5c).

### The role of Fasudil and FSD-C10 on neurotrophic factors

**Figure 6:**
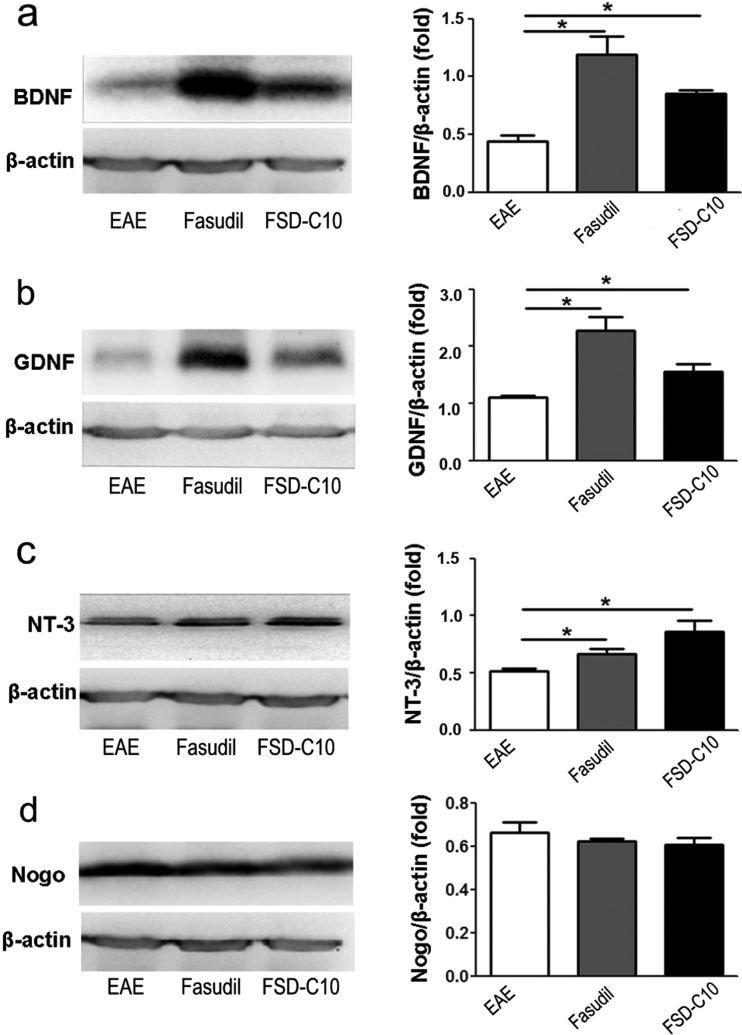
The effect of Fasudil and FSD-C10 on neuronal growth factors Chronic EAE was induced in C57BL/6 mice with MOG_35–55_ and treated with Fasudil or FSD-C10. On day 28 p.i., brains were harvested to prepare protein for the expression of BDNF (**a**), GDNF (**b**), NT-3 (**c**) and Nogo (**d**) by Western blot. The results were expressed as the fold change relative to β-actin as the loading control. Quantitative results are mean±S.E.M. of six mice in each group. *P<0.05.

Previous data suggest that the neurotrophic and neuroprotective molecules directly or/and indirectly contribute to neurite outgrowth of neurons. In the present study, we explored the role of Fasudil and FSD-C10 on production of the neurotrophic factors *in vivo*. Administration of Fasudil or FSD-C10 significantly enhanced expression of BDNF, GDNF and NT-3 proteins in brain compared with control EAE mice (Figures 6a–6c, all *P*<0.05). In contract, Fasudil or FSD-C10 administration slightly, although not significantly, declined the expression of Nogo protein level in brain (Figure 6d). These results reveal that the neurite outgrowth of neurons and dendritic formation of BV-2 microglia could be related to increased neurotrophic factors.

### The role of Fasudil and FSD-C10 on the polarization of microglia/macrophages

**Figure 7:**
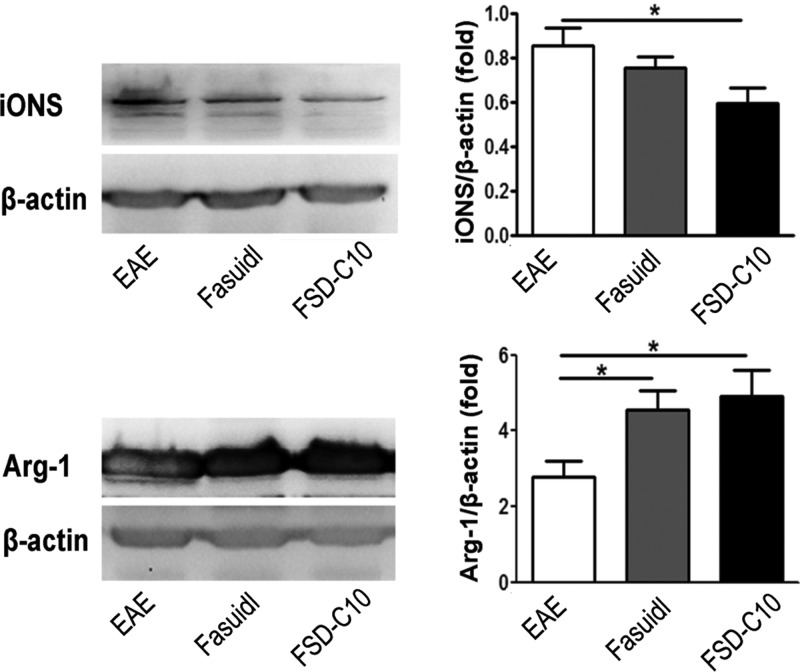
Fasudil and FSD-C10 shifted M1 to M2 phenotype Chronic EAE was induced in C57BL/6 mice with MOG_35–55_ and treated with Fasudil or FSD-C10. On day 28 p.i., brains were harvested to prepare protein for the expression of iNOS and Arg-1 by western blot. The results were expressed as the fold change relative to β-actin as the loading control. Quantitative results are mean±S.E.M. of six mice in each group; *P<0.05.

The classical M1 polarization of macrophages/microglia has been linked with promoting inflammation, whereas the M2 phenotype is anti-inflammatory and promotes tissue repair [1]. Our previous studies clearly demonstrated that Fasudil can convert M1 microglia/macrophages to M2 cells [5,6]. iNOS and Arg-1 are two representatives of M1 and M2 microglia/macrophages respectively and we thus measured their expression in brain of EAE treated with Fasudil or FSD-C10. The results showed that FSD-C10, like Fasudil, inhibited iNOS expression and elevated Arg-1 expression in brain of EAE mice (Figure 7), indicating that FSD-C10, like Fasudil, also converts inflammatory M1 cells toward anti-inflammatory M2 cells *in vivo*, which is consistent with the decrease in inflammatory cytokines described above.

### The influence of Fasudil and FSD-C10 on vasodilation and mortality

**Figure 8:**
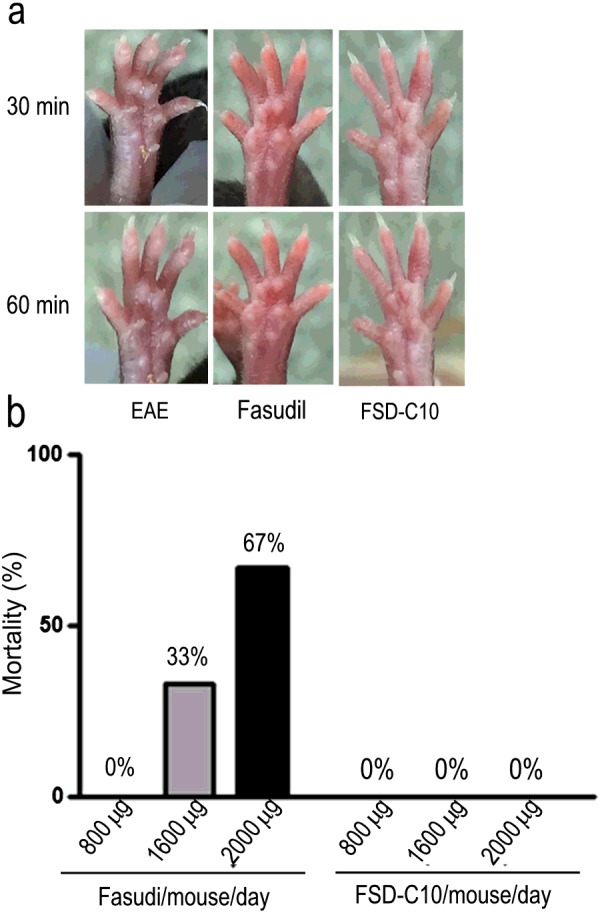
The influence of Fasudil and FSD-C10 on vasodilation and mortality (**a**) After i.p. injection of FSD-C10 or Fasudil, we observe the vasodilation of mice limbs was observed after 30 and 60 min. A representative of three mice each group is shown. (**b**) After i.p. injection of FSD-C10 or Fasudil at 800, 1600 and 2000 μg/mouse, animal mortality was recorded with 2 h.

The vasodilation and the fluctuation of blood pressure is a side effect and thus limits the long-term use of ROCK inhibitor Fasudil in the nervous system diseases, especially in chronic progressive disorders. We thus compared the role of Fasudil and FSD-C10 on the vasodilation in healthy mice. The results showed that an obvious vasodilation after 30 and 60 min on the foot of mice was observed in mice that were i.p. injected with Fasudil (800 μg/mouse) compared with mice injected with FSD-C10 at the same dose (Figure 8a), revealing that FSD-C10 has a relatively weak vasodilator effect in mice. Simultaneously, the injection of Fasudil (1600 and 2000 μg/mouse/i.p) caused nearly 33%–67% mortality within 2 h, whereas none of the mice injected with FSD-C10 died (Figure 8b). Given that FSD-C10 was well tolerant *in vivo*, further pre-clinical studies are valuable for the treatment of MS.

## DISCUSSION

Based on two reasons: (1) ROCK inhibitor should be a promising drug target for preventing neurodegeneration and stimulating neuroregeneration in stroke patients and animal models of stroke, MS, ALS, AD and PD and (2) ROCK inhibitor Fasudil in clinical practice exhibits the following limitations, including a relatively narrow safety window, not suitable for long-term use and poor oral bioavailability, researchers are looking for novel ROCK inhibitors that are more efficient, safer, oral and for long-term use for the treatment of neurological disorders. Our previous studies have demonstrated that a novel ROCK inhibitor FSD-C10 ameliorated the clinical severity of EAE, accompanied by improvement of demyelination and the inhibition of inflammatory cells in the CNS of EAE mice, exhibiting the therapeutic effect in EAE [20]. In the present study, we further compared therapeutic potential, the inhibitory efficiency of ROCK, the cell cytotoxicity, neurite outgrowth and dendritic formation, neurotrophic factors and vasodilation between Fasudil and FSD-C10. Our results first demonstrated that FSD-C10 and Fasudil exerted comparable effect in ameliorating the clinical severity of EAE. Both Fasudil and FSD-C10 induced neurite outgrowth of primary neurons and dendritic formation of BV-2 microglia as well as the production of neurotrophic factor BDNF, GDNF and NT3. Importantly, FSD-C10 treatment in cultured cells and in healthy mice exhibited relatively low cell cytotoxicity and vasodilation respectively, as compared with Fasudil, thus being safer for clinical application.

Consistent with previous findings that the inhibition of ROCK contributes to the neurite outgrowth of neurons [18], our results also showed that both Fasudil and FSD-C10 induced neurite outgrowth of neurons, as well as dendritic formation of BV-2 microglia. The neurite outgrowth is not only a result of a simple expansion and proliferation of neuronal cells, but also entails the neuronal plasticity that is highly dependent on the microenvironment, in which neurotrophic factors promote neurite outgrowth from cultured adult rat dorsal root ganglion (DRG) neurons [21]. One of them is an involvement of guidance molecules that attract or repulse growing neurite depending on the nerve cell and receptor types. In our study, the expression of three neurotrophic factor BDNF, GDNF and NT-3 was induced by Fasudil or FSD-C10, revealing that neurotrophic factor signalling axis may be a mechanism in neurite outgrowth of primary neurons and dendritic formation of BV-2 microglia.

BDNF is a neurotrophin involved in neuronal survival, differentiation and function, axonal growth and dendritic plasticity in the CNS [22]. BDNF was shown to exert neuroprotective effects in several experimental models of neurological diseases [23–25]. The importance of BDNF for brain function and maintenance is underscored by the fact that BDNF-deficient mice already die during the first weeks of life [26]. Even heterozygous mice with 50% reduced levels of BDNF expression display some behavioural deficits such as aggressiveness, hyperactivity, hyperphagia and obesity [27–29]. GDNF has also been found to be involved in a considerable number of effects in the nervous system, including the survival, migration, differentiation and neurite outgrowth of neurons [30]. NT-3 is a growth factor that modulates glial cell biology and myelination in the CNS and promotes oligodendrocyte precursor proliferation, survival and differentiation [31–33]. Further, NT-3 has significant capacity to provide neuroprotection and reduce astrogliosis, which is an important mechanism underlying the formation of MS plaque [34]. When applied to spinal cord injury, NT-3 overexpression promoted remyelination, axonal regeneration and functional recovery [25]. Following transplantation of NT-3 transduced neural stem cells, NT-3 probably acted not only on donor cells in an autocrine fashion, but also on host cells (paracrine) to enhance neuronal differentiation of both transplanted and endogenous cells and effectively suppressed EAE [35]. In addition, mouse helper T 2 (Th2), but not Th1 cells, expressed a high-affinity receptor for NT-3 and adding NT-3 to cultured Th2 cells to produce a large amount of Interleukin 4 (IL-4) [36], suggesting a direct link between immunomodulation, neuroprotection and therapeutic effects in CNS inflammatory demyelination [37]. Taken together, a set of experiments clearly demonstrate that neurotrophic factor BDNF, GDNF and NT-3 are able to provide excellent environment for neurite outgrowth of neurons that will be ultimately useful for the neural repair and regeneration purposes [38,39].

The role of the Rho/ROCK/LIM domain kinase (LIMK) pathway in neurite outgrowth has been addressed in numerous studies, but the specific contribution of single members of this cascade remained insufficiently understood. Previous studies indicated the inability of adult mammalian CNS axons to regenerate after injury, partly due to the presence of myelin-associated neurite outgrowth inhibitors, including Nogo, myelin-associated glycoprotein (MAG) and MOG. These molecules combine to the same receptor complex and activate downstream signalling molecules, causing the inhibition of neurite growth [40,41]. Rho and ROCK have been identified as downstream signalling molecules of these neurite growth inhibitors and are key elements for neurite growth inhibition and growth cone collapse elicited by these inhibitors [42]. In the present study, the inhibition of ROCK by Fasudil or FSD-C10 slightly, not significantly though, declined the expression of Nogo. Further studies are necessary to fully evaluate the role of FSD-C10 in the neuroprotection and neuroregeneration.

In addition, ROCK appears to mediate the vasoconstrictor effects and is involved in the regulation of nitric oxide (NO) pathway [43], thus contributing to the maintenance of basal vascular tone [44] and possibly participating in the pathogenesis of human hypertension [45]. Both ROCK I and II are highly homologous and share more than 20 immediate downstream substrates [46]. Two isoforms of ROCK I and II are expressed in vascular smooth muscle cells and inhibit myosin light-chain phosphatase (MLCP) activity [47], whereas ROCK I appears to play a predominant role in vascular inflammation [48]. Additionally, a critical role for ROCK I in mediating cardiac fibrosis was also observed [49]. The activation of Rho/ROCK I signalling disrupts the translocation of occluding/zona occulden-1 (ZO-1), which are the major components of endothelial tight junction. Therefore, we hypothesize that the insensitivity of vascular dilatation in FSD-C10-injected mice may be related to a relatively weak inhibition of ROCK I compared with Fasudil, thus avoiding potential side effect of the latter. Indeed, the role of FSD-C10 in relation to bioavailability, subsequent vascular remodelling and therapeutic potential is still a worthy of further investigation.

## Abbreviations

AD: Alzheimer's disease
ALS: amyotrophic lateral sclerosis
Arg-1: arginase-1
BDNF: brain-derived neurotrophic factor
CNS: central nervous system
ddH_2_O: double-distilled water
EAE: Encephalomyelitis
GDNF: glial cell line-derived neurotrophic factor
i.p.: intraperitoneally
LDH: lactate dehydrogenase
MOG: mouse myelin oligodendrocyte glycoprotein
MS: multiple sclerosis
NT-3: neurotrophin-3
p.i.: post-immunization
PD: Parkinson's disease
ROCK: Rho kinase
