# Expression of Concern: FSD-C10: A more promising novel ROCK inhibitor than Fasudil for treatment of CNS autoimmunity

**DOI:** 10.1042/BSR-2015-0032_EOC

**Published:** 2022-11-11

**Keywords:** Fasudil, FSD-C10, Rho kinase, Rho kinase inhibitor

The Editorial Office has contacted the authors of this *Bioscience Reports* article with regards to two of the images, Figures 5 and 6. A similar region of the Fig.5C Fasudil (5ug/ml) panel has been noted in the FSD-C10 (15ug/ml) panel (region rotated 180 degrees), along with similar bands being identified in the Fig.6 Western blots (the EAE and Fasudil lanes of panels 6A and 6D appear as the Fasudil and FSD-C10 bands of 6C). The authors have cooperated with the *Bioscience Reports* Editorial Office and have offered explanations for the duplicated regions, including that one of the lanes was erroneously removed from the western blots whilst compiling the figures. They have stated that the duplicated region (and image reversal) for Figure 5 could also have been due to errors whilst putting together the figures. The authors are currently searching for the original data to correct the images; however, given the age of the article, they have yet to be able to source the data. The authors state that the results of their experiments are unaffected by these issues, and as no further issues have been identified by the Editorial Office, the article will remain published with an Expression of Concern to alert readers to the duplicated regions.

